# Health and climate related ecosystem services provided by street trees in the urban environment

**DOI:** 10.1186/s12940-016-0103-6

**Published:** 2016-03-08

**Authors:** Jennifer A. Salmond, Marc Tadaki, Sotiris Vardoulakis, Katherine Arbuthnott, Andrew Coutts, Matthias Demuzere, Kim N. Dirks, Clare Heaviside, Shanon Lim, Helen Macintyre, Rachel N. McInnes, Benedict W. Wheeler

**Affiliations:** School of Environment, University of Auckland, Private Bag 92019, Auckland, 1142 New Zealand; Department of Geography, University of British Columbia, 1984 West Mall, Vancouver, BC V6T 1Z2 Canada; Centre for Radiation, Chemical and Environmental Hazards, Public Health England, Chilton, OX11 0RQ UK; European Centre for Environment and Human Health, University of Exeter Medical School, Knowledge Spa, Royal Cornwall Hospital, Truro, Cornwall TR1 3HD UK; Department of Social and Environmental Health Research, London School of Hygiene and Tropical Medicine, 15-17 Tavistock Place, London, WC1H 9SH UK; School of Earth, Atmosphere and Environment, Monash University, Melbourne, Victoria 3800 Australia; Cooperative Research Centre for Water Sensitive Cities, Australia; Department of Earth & Environmental Sciences Physical and Regional Geography Research Group - Regional climate studies Celestijnenlaan 200E, KU Leuven, 3001 Heverlee (Leuven), Belgium; School of Population Health, University of Auckland, Private Bag 92019, Auckland, 1142 New Zealand; Met Office Hadley Centre, FitzRoy Road, Exeter, Devon EX1 3 PB UK

**Keywords:** Street trees, Ecosystems services, Health impacts, Climate

## Abstract

Urban tree planting initiatives are being actively promoted as a planning tool to enable urban areas to adapt to and mitigate against climate change, enhance urban sustainability and improve human health and well-being. However, opportunities for creating new areas of green space within cities are often limited and tree planting initiatives may be constrained to kerbside locations. At this scale, the net impact of trees on human health and the local environment is less clear, and generalised approaches for evaluating their impact are not well developed.

In this review, we use an urban ecosystems services framework to evaluate the direct, and locally-generated, ecosystems services and disservices provided by street trees. We focus our review on the services of major importance to human health and well-being which include ‘climate regulation’, ‘air quality regulation’ and ‘aesthetics and cultural services’. These are themes that are commonly used to justify new street tree or street tree retention initiatives. We argue that current scientific understanding of the impact of street trees on human health and the urban environment has been limited by predominantly regional-scale reductionist approaches which consider vegetation generally and/or single out individual services or impacts without considering the wider synergistic impacts of street trees on urban ecosystems. This can lead planners and policymakers towards decision making based on single parameter optimisation strategies which may be problematic when a single intervention offers different outcomes and has multiple effects and potential trade-offs in different places.

We suggest that a holistic approach is required to evaluate the services and disservices provided by street trees at different scales. We provide information to guide decision makers and planners in their attempts to evaluate the value of vegetation in their local setting. We show that by ensuring that the specific aim of the intervention, the scale of the desired biophysical effect and an awareness of a range of impacts guide the choice of i) tree species, ii) location and iii) density of tree placement, street trees can be an important tool for urban planners and designers in developing resilient and resourceful cities in an era of climatic change.

**Electronic supplementary material:**

The online version of this article (doi:10.1186/s12940-016-0103-6) contains supplementary material, which is available to authorized users.

## Background

Urban tree planting initiatives are being actively promoted as an urban planning solution to reduce the environmental degradation caused by urbanization, enhance urban sustainability, mitigate and adapt to climate change and to improve human health and well-being [1, 2]. The public perception of the value of green spaces and green infrastructure (especially trees) within cities has prompted a number of initiatives to promote the ‘greening’ of cities through urban reforestation and protection programs to increase the percentage of tree canopy cover, such as the New York City ‘Million Trees’ program [3], or the City of Melbourne’s 40 % tree canopy cover target. Such projects have stemmed from a wide range of different organisational bodies encompassing local to international-scale governance, community based, charitable and regulatory approaches. Here, the broader arguments for increased tree density stem from benefits for public health and quality of life, and the sustainability and resilience of cities in light of climate change [4].

However, two issues immediately arise. First, opportunities for urban greening remain limited in cities. Land is expensive and trees require economic and environmental resources to survive as assets in the harsh environmental conditions characteristic of urban areas. Careful thought needs to be put into considering their placement, their beneficiaries, viable alternatives, who is responsible for ongoing costs and maintenance, and potential co-benefits with urban planning objectives at multiple scales. Second, urban trees do not provide ubiquitous ‘good’ for all actors in all contexts. The complex physiology and ecological functioning of trees mean that efforts to optimise for one ‘good’ (such as less leaf litter or shade) can produce undesirable effects (such as increased aero-allergens) for different sites, scales and social groups. Thus, key questions remain in urban design and planning as to how to invest in green urban infrastructure in ways which incorporate the large body of scientific understanding of multiple biophysical and social processes in ways relevant to human decision making.

The application of urban climate, environmental and social sciences in this field is in its infancy, and few studies have sought to integrate understanding of the physical world with the social and cultural contexts of urban environments. Given the heterogeneity and complexity of the processes which determine the environmental and social impacts of urban vegetation, it is not surprising that there have been few attempts to synthesise the current knowledge about the net impact of trees on the physical, public health and cultural aspects of the urban ecosystem. Current research in this field often emphasises a singular benefit and direct planners towards a single-variable optimisation strategy. This becomes problematic when a single-variable intervention offers different outcomes and has multiple effects and potential trade-offs. For example, current preference for male over female trees of the same species in many North American and European cities to reduce mess from seeds and fruit can result in higher pollen loads in the atmosphere [5].

There is a pressing need for holistic assessments of the health impacts of climate change mitigation/adaptation policies such as the promotion of street trees. Vegetation provides shade and humidity thereby reducing surface and air temperatures at local scales and thus is a potential adaptation strategy in an era of climate warming. Given that increasing vegetation density also has the potential for significant co-benefits to be realised across a range of public health arenas, exploring the two themes of health and climate enables a broader appreciation of the complexity of the issues and services realised at different scales in different urban settings. We focus on trees along streets, as street trees represent a particular mode of greening urban areas which offer particular services and functions [6, 7]. As such, there is significant interest in the potential of street trees as a tool in urban design to mitigate against a number of climate-related urban problems.

This paper provides a critical review of the potential of street trees as an urban planning (or engineering) solution to improve human health and well-being through ‘climate regulation’, ‘air quality regulation’ and ‘aesthetics and cultural services’. These are themes that are commonly used to justify new street trees or street tree retention initiatives. We seek to match changes in these biophysical processes resulting from street trees with health impacts (such as physical health, mental health and the well-being of residents) at relevant scales.

We utilize an urban ‘ecosystem services’ (ESS) framework [4, 8] as a platform through which to synthesize current knowledge, and assess the holistic value of street trees by thinking through the different processes and functions that street trees perform which are of human value in the spheres of climate and health. While most ESS typologies often present the potential climate, air quality and cultural-aesthetic benefits of trees in a ‘list’ fashion, these are rarely discussed in sufficient detail to highlight contradictions and the place-specific context of results. We identify the limitations of promoting investment rationales for street trees drawn from single-issue modelling studies that highlight a single benefit or even co-benefit (e.g. Jim and Chen [9]). This leads us to propose some methodological recommendations about how the impact of street trees on urban ESS could be approached differently, and how future analyses might be oriented to facilitate dialogue about the diverse meanings of trees and green space in urban environments.

### An urban ecosystem services approach (ESS)

Much research and advocacy has focussed on documenting the human benefits arising from integrating various forms of ecological restoration (such as urban tree-planting) into urban design and planning [10, 11]. The ‘ecosystem services’ approach is increasingly being utilized by researchers, advocates and policy makers to highlight and evaluate the human benefits received through the ecological functioning provided by urban trees and other such ‘ecological infrastructure’ [4, 10, 12]. Ecosystem services refer to the subset of ecological functions that are directly or indirectly linked to human benefits or well-being [13]. What is crucial about the ecosystem services framework is that it analyses the relationships between specific ecological processes and attributes, and specific outcomes of value to humans. Analytically, this means focussing on identifying, quantifying and modelling the human benefits (and costs) of ecological and biophysical processes relating to urban green infrastructure.

What constitutes ‘best practice’ in identifying and classifying ecosystem services (ESS) has been debated, contested and refined over the years for various purposes [14, 15]. In mainstream ESS thought, a four-part typology of services distinguishes: provisioning services (direct outputs of human value, such as food), regulating services (maintenance of valuable processes, such as water purification by wetlands), supporting services (processes indirectly valued, such as pollination) and cultural services (providing valued social and spiritual meanings) [16]. Some scholars have developed more specific classifications of ESS for urban environments. One study [12] provided an early and simple categorization of ESS unique to urban ecosystems and environments, highlighting how urban green infrastructure provides benefits to human health in the forms of micro-climate regulation, air filtration, noise reduction, rainwater drainage, sewage treatment and cultural values. Another [10] expanded this typology and situated a range of urban ESS underneath each of the four major classes used in the Millennium Ecosystem Assessment (see Table 1).

**Table 1:** Urban ecosystem services relevant to human health. Classification adapted from [8]

Service class	Specific services
Provisioning services	Food supply, water supply
Regulating services and related health benefits	Urban temperature regulation, noise reduction, air quality improvement, moderation of climate extremes, runoff mitigation, waste treatment, pollination, pest regulation, seed dispersal, global climate regulation
Supporting (habitat) services	Habitat for biodiversity
Cultural services	Recreation, aesthetic benefits, cognitive development, place values and social cohesion

While urban ESS classifications and lists of the environmental services and disservices provided by street trees (provided in reviews elsewhere [2]) provide useful heuristics for highlighting the *potential* services provided by urban ecological infrastructure, detailed reviews are needed to assess the weight of evidence, contextual variability and robustness of the relationships that have been documented linking *specific* urban design elements to *specific* human benefits in particular urban contexts.

This review embraces the ESS framework to critically review the literature pertaining to the potential benefits of street trees for urban design and human well-being. We view street trees as a specific ‘ecosystem component’ involved in the delivery of services [17]. As noted in the Introduction, street trees are increasingly viewed as a planning solution to urban problems; they are being included as integral components for climate sensitive urban design, for urban liveability and environmental justice [6]. By critically reviewing the scientific literature for a range of often-proposed ESS for street trees, we aim to inform and advance dialogue in urban planning about the role/s that street trees might play in pursuing a range of societal objectives.

We use the ESS framework to organize our review around the services (and disservices) provided by street trees, emphasising the regulating and supporting services identified by Gomez-Baggethun et al. [10] which are relevant at local scales to climate mitigation and human health. However, the framework also brings into focus three further points. First, it has been well acknowledged that much ESS work is reductionist, in that it focusses on one or two elements or services (such as climate regulation provided by trees) ignoring other functions or processes of potential value to humans. It has been argued that ESS has become a ‘complexity blinder’ [18] that conceals as much as it reveals about which ecological processes (should) matter to humans. Second, while we take street trees as a useful starting unit for analysis, the ESS literature sensitizes us to the scale-dependent provision of services [1]. That is, the benefits provided by a unit of street trees may be dependent upon whether street trees and/or other related green infrastructure are providing similar services nearby. Third, and relatedly, the ESS framework highlights how ‘benefits’ are social constructs that are context specific [19]; what is beneficial in one context may not be in another, and what is seen as ‘beneficial’ by one social group may not be seen as beneficial by another. In summary, ESS analyses need to be grounded in their particular biophysical and social contexts; our review attends to these insights as relevant for street trees.

We also draw on the cultural ecosystem services literature as a framework for thinking about the diverse ways in which street trees are meaningful to human subjects [1]. We approach cultural ecosystem services broadly as the “contributions of ecosystems (or nature) to human well-being via nonmaterial connections” [20]. This definition emphasizes the importance of *meaning* to human actors (i.e. the ‘nonmaterial connections’). This aspect is important from a human well-being point of view, but is less tangibly connected to notions of physical environment.

The following sections provide a discussion of a selection of the relevant literature to highlight the challenges associated with determining the impact of street trees both on the local-scale physical processes operating within urban ecosystems and also the social, cultural and health aspects. The literature on these topics is vast. We have been very selective in our use of case studies and examples and do not claim to provide an exhaustive review or systematic list of all services and disservices (see Roy and Pickering [2] for this). Rather we are performing a wider information-organizing function for prospective decision makers to help make sense of 1) the diversity in ESS for urban street trees, as well as 2) the importance of tree species, density and location in service provision for any given location, and 3) the implications and potential health and societal effects of optimising for a singular service.

### The role of street trees in provision of regulating services

#### a) Micro-climate

As a result of the extensive replacement of natural soils and vegetation with impervious surfaces, cities have warmer drier climates than their rural counterparts at local, urban and regional scales, especially at night [21]. Increasing vegetation cover in urban areas leads to reduced ambient and surface temperatures and increased evapotranspiration, precipitation interception and reduced runoff. Increasing the vegetation density is therefore considered an effective option for mitigating urban heat and thereby adapting to climate changes caused both by regional-scale changes in land use and global-scale changes in atmospheric composition [22]. However, little is known about the general effects of changing the density of street trees on urban climates at regional or local scales.

Most studies of heat effects on health are undertaken at regional scales and use mean daily temperature or maximum daily temperature as the most relevant predictor for mortality or morbidity [23–25]. From a health perspective, urban residents are particularly at risk of suffering from heat stress, especially during extreme heat events as locally generated heat exacerbates the effects of regional scale heatwaves [26]. Typically, urban climate modelling studies at similar scales employ urban land surface schemes which categorise vegetation cover generally rather than specifically street trees. Such studies do show that increased vegetation cover results in reducing both mean air temperatures [27, 28] and extreme temperatures during heat waves [29]. Some studies have also shown that the cooling effect of vegetation at a regional scale is more pronounced at night [29]. This is significant from a health perspective since minimum temperature has also been strongly associated with mortality due to the inability of the body to recover from heat stress during the night time period [30].

Where predicted temperature changes have been related to changes in health parameters, simple statistical correlations are often used which cannot easily be applied in other contexts. For example, it has been found that a 20 % increase in vegetation cover resulted in a 7.18 % decrease in 24-h average temperature in Phoenix, Arizona, where hot dry conditions dominate [31]. This was then projected to reduce average annual heat-related emergency calls by 11 % [31].

While such regional-scale research highlights the potential mean temperature reduction from increasing vegetation, modelling studies generally employ a resolution of around 1-5 km and are unable to capture the type of vegetation or exactly where it is placed (e.g. parks or street trees). This general approach to representing ‘vegetation’ may therefore bias results and not prove accurate for predicting the local effect of street trees. In one rare study of the impact of increasing just street trees on temperatures at these urban to regional-scales [32] showed only a very small reduction in the average air temperature at 1500 h of between 0.2 and 0.5 °C during heat waves in New York City. However, again, the results are specific to the local characteristics of urban form and general climate zone.

To understand the underlying processes which relate changes in tree cover to changes in climate, local-scale processes need to be characterised and understood. Trees provide shade, blocking solar radiation from reaching pedestrians [33] and limit solar heating of impervious surfaces with high heat capacity and thermal conductivity (such as concrete), reducing heat storage. Vegetation can increase urban albedo (compared to dark asphalt surfaces), and vegetated surfaces have lower radiative temperatures than impervious surfaces with the same albedo [34, 35].

At local scales, extensive tree coverage can deliver significant benefits to outdoor human thermal comfort (a measure of the temperature and humidity of the environment in relation to the body’s ability to maintain a comfortable core temperature) and result in lower heat stress levels [36, 37], especially during extreme heat events [38]. At these scales, the changes in temperature observed from the presence of street trees can be much larger than regional effects, but are highly variable and difficult to generalise. For example, in Bangalore, India, an experimental study showed that afternoon ambient air temperatures were 5.6 °C lower in roads lined with trees, and road surface temperatures 27.5 °C lower than those measured in comparable tree-less streets [39]. Observations from a courtyard in Israel with shade trees and grass showed reduced air temperatures of up to 2.5 °C [40]. The impact on local climate is dependent on the prevailing regional climatic context, geographic setting of the city, urban form, the density and placement of the trees, species type, age and the health of the tree.

However, even when average air temperature reductions from street trees are small, the net benefits of trees from shading effects for human thermal comfort can be substantial. Shading is critical for improving human thermal comfort, particularly via reductions in mean radiant temperature which is the dominant influence on outdoor human thermal comfort under warm, sunny conditions [40, 41]. Shashua-Bar and Hoffman [34] also note that within the urban canyon, as much as 80 % of cooling from trees comes from shading.

The presence of street trees can also modify indoor temperatures by shading buildings and significantly reducing the risk of indoor overheating) [42]. This can benefit human health where economic resources are unavailable to cool buildings or could provide further co-benefits by reducing energy demands for building cooling [43]. One study shows that tree shade can reduce wall temperatures by 9 °C and air temperatures by up to 1 °C [44]. It also argues that it is very difficult to generalise the impact of trees on building thermal performance as there is very limited data available and the impacts are dependent on materials, architecture and design, geometry, tree species, aspect and season.

However, the positive summertime effects of street trees during the daytime need to be counter-balanced by their night and wintertime impacts. At night, although the presence of trees may reduce local-scale heat storage and hence release at night, street trees trap radiation within the canyon and reduce ventilation, preventing the dissipation of sensible heat that has built up during the day. Therefore, while an extensive tree canopy cover may be beneficial during the day, there is a risk of restricted nocturnal longwave cooling leading to slightly higher and more uncomfortable indoor temperatures during the night [38]. It should also be noted that trees change aerodynamic resistance to heat diffusion, and may limit the penetration of breezes and cooling of buildings through open windows at night during summer.

While the health effects of increased heat are damaging, the majority of deaths caused by temperature in urban areas around the world are associated with moderately cold weather rather than heat [25, 45, 46]. Therefore a drop in ambient temperature during the winter caused by shading from ever-green street trees could have a negative effect on health. Reduced light levels in the winter time could also have an impact on mental health for individuals sensitive to Seasonal Affective Disorder [47]. Increased shading can also result in lower indoor temperatures, increasing mould and dampness within buildings and increase energy consumption for building heating in winter.

There is a synergistic relation between trees and climate. Water has an important role to play in maintaining full and healthy, actively transpiring tree canopies. Urban environments can place additional pressures on street trees [48] that may not be experienced by their rural ‘forest tree’ counterparts. Elevated urban temperatures, dry air and soils and large radiative loads (especially on isolated street trees) can lead to a very high evaporative demand [49, 50]. Without alternative irrigation sources to increase soil moisture and support street trees, as well as to dissipate high heat loads [51], their health and capacity to cool urban environments can be impaired. This could be particularly significant in many urban areas given projected climate change patterns.

Trees generally increase humidity, acting as channels for water loss to the atmosphere [51] with their roots drawing moisture from deeper layers of the soil. Water sensitive urban design, storm water harvesting and recycled water can all provide a means for increasing soil moisture levels in cities where water availability is an issue. Biofiltration systems and irrigation from rainwater tanks can deliver substantial increases in evapotranspiration as a result of stormwater retention [52]. Such measures have additional eco-hydrological benefits including reducing run-off (which benefits downstream waterways), and improving soil drainage and soil erosion control [53]. Street trees intercept and store rainfall, filter runoff in the canopy and in the root-zone, and draw moisture from the soil, increasing the soil water storage capacity for rainfall events [54]. Trees also modify the below-ground environment, improving the permeability of soils [55]. In these ways, indirect health benefits from reduced flooding and storm water damage can be achieved. However, these effects are difficult to quantify [1].

In summary, there is some evidence to support the notion that increasing vegetation density in urban areas can lead to positive changes from both the local climate and health perspectives. However, most studies linking climate variables to health have been undertaken at regional scales, and little is known about the underlying biophysical processes or causal pathways which specifically link street trees to health effects at local scales. Thus, as demonstrated in the next sections, the evidence for the direct effect of street trees on health remains poor. Although at local scales the effects of street trees on climate and hence human health is context specific, some generic recommendations can be made when just considering direct climate effects and health. For example, during the day, street trees tend to be more effective in cooling streets which are exposed to large amounts of solar radiation (wide open streets of low height-to-width (H:W) ratios [56] and those oriented east-west [57]). As the H:W ratio increases, the role of building shade and thermal mass begins to overwhelm the contribution of street trees in cooling [38]. Clustering trees into lines or small groups [58] interspersed with open areas in a ‘savannah’-type arrangement [59] can help reduce the radiative load [51], provide shade, and allow longwave cooling at night. Large, wide trees with dense canopies could be considered for streets with low H:W, while taller narrower trees could be considered for streets with high H:W. However, uncertainty remains in the literature, as it has been suggested that the cooling effects of trees is related mostly to planting density and canopy coverage [56], while others note that attributes of tree species like leaf colour and leaf area index can also strongly influence cooling [60].

#### b) Air quality and noise regulation

The potential impact of street trees on air quality remains one of the most poorly understood aspects of the studied ecosystem services and benefits [61]. Street trees have the potential to regulate air quality by absorbing pollutants and increasing pollutant deposition. They emit pollutants and pollutant precursors in the form of biogenic volatile organic compounds and pollen and may also regulate the soundscape of the city. However, the plethora of processes operating at different scales make it very difficult to predict the net effect of street trees on air quality in any given environment. The ESS framework is important here in assisting with matching scales of study with outcomes.

#### c) Deposition and dispersion

The health effects of air quality regulation by trees in the urban environment have mainly been studied at regional scales using modelling approaches which have not been extensively validated with field trials. Most studies at regional or city scales show a modest modelled reduction in pollution concentration of less than 5 % resulting from urban vegetation [62, 63]. Trees increase both the surface roughness (slowing air flow thus enhancing deposition and absorption pollutant removal processes) and the area of the ground surface that atmospheric pollutants come into contact with (acting as biological filters, enhanced by the properties of their surfaces) [64]. Trees absorb CO_2_ and gaseous pollutants such as O_3_, NO_2_, SO_2_ primarily by uptake via leaf stomata or surface, and accumulate airborne particulates (by interception, impaction or sedimentation) more effectively than other urban surfaces [65–67].

Estimates of the resulting modelled improvements in air quality from vegetation are generally extrapolated at regional scales in association with health metrics using large-scale epidemiological approaches, and few studies specifically focus on urban greening. For example, it has been suggested current woodland cover (non-urban) in Great Britain mitigates between five and seven deaths and four and seven hospital admissions annually due to reduced PM_10_ and SO_2_ concentrations [68]. However, similar to the pitfalls associated with assigning a monetary value to the economic benefits of street trees [69, 70], such calculations are dependent on the accuracy of the underlying assumptions used in the methodological approaches.

At local scales there is little evidence to link air quality regulation from vegetation with improved health outcomes. Indeed at local scales, studies are less conclusive as to the direction of the relation between vegetation and pollution, possibly because the interplay between urban form and vegetation becomes important. At local scales, the characteristics of the tree canopy, tree density and proximity to other urban structures influence the ability of plants to remove pollutants [71, 72]. The rate of pollutant removal is species dependent, and trees with a large leaf surface area can remove 60 to 70 times more gaseous pollutants a year than small ones [69]. However, the extent to which particle concentrations can be reduced via deposition is more controversial, as particles can be washed off and re-suspended [73]. Besides being affected by particle size (see Janhäll [67] for a comprehensive review), plant species differ in their ability to scavenge dust-laden air due to their differing features such as habitus, canopy height, or position, size, of the morphology (shape, texture, roughness) of leaves (e.g. [62, 72, 74, 75]).

At local scales, changes to the urban air flow regimes from the tree canopy may also reduce the horizontal and vertical exchange of both clean and polluted air between the urban canyon and its surroundings (also referred to as the ventilation hypothesis [76]). Many depositional studies do not take this into account and therefore may underestimate the effective deposition rate.

Similar challenges are associated with attempts to quantify the effect of street trees on canyon-scale pollutant dispersion processes. This makes it difficult to generalise the net impact of street trees on local air pollution concentrations. A plethora of wind tunnel and computational fluid dynamics (CFD) studies have been performed on idealized urban geometries with trees to characterise the under-lying processes which determine local dispersion effects on one (see Moonen et al. [77] and references therein) or two intersecting street canyons [78–80]. Unlike the studies which focus on deposition and removal processes, most of these dispersion-led studies report a localised increase in traffic-related gaseous pollutant and particulate matter concentrations associated with increased tree cover. The results remain consistent when scaled up to neighbourhood areas with one study [81] reporting an increase in average pollutant concentrations of 1 % associated with every 1 % increase in tree crown volume fraction relative to the tree-free situation for occupation fractions of 4-14 %. It is therefore unclear to what extent this impact of street trees on air quality remains valid for ‘real’ street canyons. In a combined modelling and field study, one study concluded that excluding the effect of vegetation results in non-negligible errors in pollutant predictions and resisted attempts to generalise the local impacts of trees on air quality [78].

A limited number of experimental studies have attempted to quantify the net change in pollutant concentrations resulting from street trees (e.g. [76, 82–84]). The results from these studies provide mixed answers as to whether trees provide a net benefit in regulating air quality, pointing to local factors as important determinants of the local effects. For example, a seasonal investigation of six street canyons in residential Shanghai (China) revealed that in the presence of street trees, the rate of decrease in concentration of PM_2.5_ with height was much lower compared to tree-less streets [85]. In comparison, another study showed that sections of major highways in Queens New York (USA) which had trees planted perpendicular to the street had fewer spikes in PM_2.5_ concentration but higher mean background concentrations, indicated reduced dispersion compared to grass-covered sections [86]. But, while trees which form a continuous tunnel or canopy within a street promote pollutant storage of pollutants emitted within the canyon, they can also reduce transport of pollutants from other locations within the city.

One study has examined experimentally the impact of street trees on indoor air quality by temporarily installing a line of young trees (silver birch) outside a row of terraced houses in a heavily trafficked street in Lancaster (UK) [87]. Their results indicated that rather than increasing total urban tree cover, single roadside tree lines of a selected, high-deposition-velocity, PM-tolerant species appear to be optimal for PM removal. However, further experimental research into vegetated streets is necessary to verify these results [88].

In summary, it remains challenging to quantify the rate of deposition using either modelling or measurement approaches. Large uncertainties remain and the ranges reported vary significantly, especially at local scales [63]. The rate of deposition also depends on the chemical species in question. For example, SO_2_ more readily deposits to surfaces (as do other acidic gases), whereas PM may be less so (and may actually be resuspended from the vegetated surface). At local scales, the specific combination of tree species, canopy volume, canyon geometry, and wind speed and direction must be accounted for on a case-by-case basis [89].

#### d) Emission of biogenic volatile compounds

Other ecosystem (dis)services associated with street trees include the direct emission of gases which act as precursors to the formation of secondary pollutants such as ozone in urban atmospheres. Trees emit biogenic volatile organic compounds (bVOCs) as a reaction to stress in their environment, such as high light intensities and/or temperatures or low water availability [90, 91]. Isoprene is the most abundantly emitted bVOC [92]. In the presence of NOx and sunlight, isoprene contributes to ozone formation, which may accumulate locally when ventilation is limited [93, 94]. Other types of bVOCs, such as monoterpenes and sesquiterpenes, are also emitted, but unlike isoprene, these continue to be emitted at night. In addition to contributing to ozone formation, terpenes can also contribute to particulate formation (Secondary Organic Aerosol – SOA) as they chemically degrade in the atmosphere [95]. Due to their very complex reactions, quantifying their contribution to pollutants is still an active area of research [96].

A recent study provides an extensive review on the emission of bVOC by street trees and their impact on O_3_ concentrations [94]. They argue that due to the limited availability of studies at the urban level, a number of key processes are still poorly understood, including the amount of bVOCs emitted by street trees, the interaction between bVOCs and urban pollution and their influence on O_3_ formation, and the effects of O_3_ on the biochemical reactions and physiological conditions leading to bVOC emissions. It should also be noted that the production of ozone from bVOC emissions may be outweighed by the reduction in ozone due to deposition and uptake by the tree, though this will depend on the specifics of the scenario. For example bVOCs from street trees may increase ozone concentrations within trafficked street canyons due to the high concentrations of NOx, but are less likely to have a significant effect in areas with low NOx concentrations.

Tree/plant species and environmental stresses (such as drought, heat, and pest infestation) influence the amount and type of bVOC emission. Temperature increase has important direct influence on rates of bVOC emissions, gas-phase chemical reaction rates, and O_3_ dry deposition, which could result in higher O_3_ levels under climate change conditions [97]. Also, here, a proper selection of tree species is relevant; a recent study indicates that planting one million low bVOC-emitting trees compared to, for example, one million English oak trees (high emitters) in Denver (USA), is equivalent of preventing emissions from as many as 490,000 cars [98]. Donovan et al [99] developed an urban tree air quality score that ranks trees in order of their potential to improve urban air quality. Of the species considered, pine, larch, and silver birch have the greatest potential while oaks, willows, and poplars can worsen downwind air quality if planted in very large numbers. To summarise, since bVOC emission (which may lead to ozone production) can vary with species, as can the effectiveness of pollutant dispersion and/or uptake, the particular tree species as well as the environment it will be sited in, need to be considered carefully to balance any benefit in pollution reduction with the potential for enhanced ozone production and altered dispersion of pollutants.

More detailed studies are required to specifically link the health effects to air quality regulation from trees at local scales. Further, although the importance of the commuter micro-environment is well known in determining personal exposure, little is known about the role of street trees in determining personal exposure whilst moving around the city using any mode of transport. Cyclists, motorcyclists and pedestrians are most susceptible to exposure to peak concentrations due to a lack of physical barrier between them and the source [100, 101].

#### e) Noise attenuation

A further atmospheric service that is often considered alongside air pollution is noise pollution. Noise in urban areas has been associated with annoyance, self-reported sleep disturbance and hypertension [102]. Little is known about the specific value of street trees in reducing noise pollution in street canyons, although there is certain evidence that trees can attenuate traffic noise roadside of open busy streets [103].

More significant is the role that urban trees may play in the masking of urban noise. Almost universally, people rate the quality of natural sounds more highly than anthropogenic sources [104]; the source of the sounds is as important as the actual intensity level. For example, the introduction of natural sounds, in urban open spaces have been shown to improve the perception of the quality of the soundscape [105–108]. While much of the focus has been on the role of water features [107], the introduction of trees within a street canyon also has the potential to significantly alter the soundscape by generating sounds associated with the rustling of leaves in response to wind, and attracting bird wildlife sounds that would be rated more positively than a street canyon dominated by road traffic noise.

#### f) Pollen

Exposure to allergenic pollen from trees is associated with a range of health effects, including allergic rhinitis, exacerbation of asthma in susceptible individuals, and eczema. These pollen grains are produced in the flowers of trees, and the timing of their release varies depending on the tree species and environmental conditions. Tree pollen is spread by the wind and its dispersion is dependent on a number of environmental factors, including the local meteorological conditions. Individuals can be sensitive to pollen from one or more different species of trees. Estimates of the levels of tree pollen allergies in the population range from around 5 % to over 50 % in Europe [109]. As such, it is a significant environmental health issue.

Some species of trees are more highly allergenic than others. Most of the allergenic tree pollen in Europe is produced by *Betula* (birch), and in Mediterranean regions, *Olea eropaea* (olive) (found mostly in agriculture rather than in cities) and *Cupressus* (cypress) [109]. Despite being highly allergenic, Betula is popular for ornamental planting in cities and streets [110]. In Europe, the largest proportion of the population with a positive skin prick test to *Betula* allergens was 54 %, recorded in Zurich, Switzerland [109]. In the city of Cordoba, Spain, *Cupressaceae* pollen accounts for 30 % of the total pollen count during winter and is responsible for allergic rhinitis at a time when no other allergenic plants are flowering [109, 111]. *Cryptomeria japonica* (Sugi or Japanese cedars) has been shown to be highly allergenic with large health effects found in populations [112, 113]. This species can be found planted in cities both in Asia and in North America. Jianan et al. [114] offer a review of allergenic planting in urban areas, with a focus on species planted in China.

The effect of interacting environmental and meteorological conditions on the production and release of allergenic tree pollen is highly complex. It is therefore unclear what effect climate change will have on pollen, although there is some evidence that it may result in earlier seasonal appearance of respiratory symptoms and longer duration of exposure to pollen [115]. The production of tree pollen is dependent not only on the current meteorological conditions (including day length, temperature, precipitation, and wind speed/direction), but also on the conditions and water availability experienced in the year prior during which pollen is formed [116]. Any changes in these conditions affect the phenology of the tree and thus the timing of the onset of pollen release, the total volume of pollen produced, and the length of the flowering season [117]. Several studies have measured the diurnal cycle of tree pollen, and have found that different species exhibit different daily cycles. Ščevková et al. [118] found that tree pollen tends to peak in the afternoon, with lowest levels observed throughout the night. Significant variations are observed between species. However, another found that Betula resulted in peaks throughout the day and night. It is unclear from the literature how the urban environment, particularly the light, water and temperature modification in streets, might affect both the timing of onset of release and the diurnal pattern of pollen release [119].

There is also a synergistic effect between pollutant concentrations and the health response to pollen. People who live in urban areas have been shown to be more affected by pollen allergies (asthma and allergic rhinitis) than those who live in rural areas [109, 120, 121]. Urban streets with high levels of vehicle emissions have been shown to coincide with increased pollen-induced respiratory allergies. There is suggestive evidence that exposure to air pollution prior to pollen exposure can exacerbate symptoms and lower the threshold of pollen required to trigger symptoms in allergy sufferers [122, 123]. To fully understand and quantify the effect of exposure to both allergenic tree pollen and traffic-related pollutants, it is necessary to determine the effect on both the allergenicity (such as increased allergenicity of pollen which had been exposed to NO_2_ found by Cuinica et al. [124]) and the volume of pollen grains released under increased air pollution. It is also important to consider the health impacts of all these factors in high co-exposure areas such as traffic-heavy urban streets. The co-exposure of pollen and air pollutants (ozone, NO_2_, SO_2_, PM_2.5_ and PM_10_) is currently an active area of research [125, 126].

In some instances there may also be a tension between the choice of tree species to mitigate air pollution and pollen production. For example London Plane Trees (*Platanus x acerifolia*) are a commonly cited source of allergy-producing pollen [127, 128], however these trees, with their large leaves, are likely to be very effective at removing pollutants from the air.

It is also important to note that, as with air quality, there are a number of feedback loops and synergistic effects which make it very difficult to predict the net effect of increasing street tree density on pollen production especially when changing climates are taken into consideration. The local effect of climate change on pollen production, release timing, transport and deposition from urban street trees is highly complex, and its impact on pollen allergies is very uncertain. Plants may release pollen earlier and for longer periods in warmer climates [122]. Increases in atmospheric CO_2_ concentration may lead to great pollen release through increased plant productivity, but plants may also be limited by other factors such as water stress.

In summary, few studies examine the complex relations between urban vegetation, urban form and air quality, especially at a local scale [8]. Thus, the trade-off between increased deposition and removal processes which act to reduce pollution concentrations against reduced horizontal and vertical dispersion, and increased biogenic (bVOC) emissions and pollen, remains poorly understood. To date, the empirical evidence available is limited in spatial and temporal extent, and is strongly dependent on case-specific local characteristics, making general conclusions difficult to justify (see Fig. 2 in Jim and Chen [8]). This is further exacerbated by the fact that street trees affect local air quality in a number of ways, driven by a complex interplay of physical and chemical processes and by variable emission sources and prevailing (urban) meteorological conditions.

### Cultural values, ecosystem services and the meanings of urban trees

Urban street trees mean different things to different people. For some, they might contribute to ‘connecting with nature’, to others, they may be a nuisance (see Roy et al. [2]). These meanings can be explored quantitatively and qualitatively, and at different scales, with different approaches making different assumptions about both the ecosystems and social groups being studied or represented. We present this section as a survey of approaches rather than as a comprehensive summary.

#### a) Quantitative approaches

Quantitative approaches to understanding the meanings of urban ecosystems for human subjects are often targeted at documenting the psychological, recreational and aesthetic benefits of natural environments to human health and well-being [20, 129, 130]. Psychological research on these topics has focused on relating access to ‘green space’ to proxies of human well-being such as self-reported levels of stress and workplace productivity [20]. Whilst the evidence is somewhat mixed, these benefits are thought to arise through mechanisms including opportunity and motivation for physical activity, stress recovery, cognitive restoration and social contact [131]. Overall, there has been limited work to date that focuses on street trees in particular (but see Schroeder et al. [132]. Tzoulas et al. [129] reviewed three dominant quantitative approaches to evaluating the relationships between urban green space and human psychological well-being outcomes: observational epidemiological studies, surveys and experimental trials.

Observational epidemiological studies have been used to examine the relationships between green infrastructure and social variables (such as human health indicators and income), using population samples and statistics to hypothesize causal relationships between them. In this context, these are often ecological in design, in other words, exposures or outcomes are aggregated at population or group level. For example, a recent ecological cross-sectional study using data for London (and controlling for other confounding variables) suggested that antidepressant prescribing rates (as an imperfect proxy for depression/anxiety amongst the local population) were slightly lower in areas with greater street tree density per length of street [133]. A different study in the Netherlands was not specifically focused on street trees, but audited ‘streetscape greenery’, and found positive associations with self-reported general health, mental health and acute health-related complaints [134]. Similarly, Lovasi et al. [135] found an inverse association between density of urban trees and the prevalence of childhood asthma (but not with hospitalisations due to asthma). Although this analysis controlled for population density, socio-economic characteristics (e.g. proportion of population living below the poverty line) and proximity to sources of air pollution, residual confounding in this study, and other observational studies, remains possible.

Practitioners in health, environmental and social sciences are increasingly mapping and investigating the spatial relationships between trees and social groups and practices, generating estimates of environmental ‘exposures’ and supporting new questions and research projects. Foremost among these could be recent work by political ecologists exploring the links between street trees and social inequality [6, 136].

Experimental studies seek to control how exposures (e.g. to street trees) are distributed across study participants in order to determine causal relationships. For example, recent laboratory-based studies exposed participants to different imagery of street scenes, with results suggesting that streets with greater tree coverage promote stress-recovery (based on standard self-report measures), although the association was non-linear [137]. A similar study suggested that this stress-recovery benefit may be gender-specific, finding a benefit only amongst men [138]. Bowler et al. [130] reviewed only experimental studies which sought to link human psychological health and the natural environment, and found a small number of generalizable relationships (e.g. positive effects on activities such as walking), calling for more rigorous experimental designs [139].

Surveys can be used to understand individuals’ interactions with – and attitudes towards – urban trees. Avolio et al. [140] surveyed five counties in California (n: 1029 surveys) about attitudes to and uses of urban trees, and revealed significant regional differences in desired tree attributes. Residents living in hotter areas value trees more for shade, and desert area residents valued trees more than those who live near natural forests. Surveys can also be used to document preferences for future desired outcomes. For example, Giergiczny and Kronenberg [7] used an economic choice modelling survey of urban residents to elicit their willingness to pay (in the form of a hypothetical tax) for planting trees in different spatial areas. They found a high willingness to pay for greening the streets in general, but the strongest preference was for greening those streets which currently have few or no trees.

A fourth quantitative approach (which we add to the three identified by Tzoulas et al. [129]) is city- or region-wide valuation studies. These use meta-data to present an administrative logic for valuing urban trees and increasing tree density. Many economic studies embrace this approach, which:

1. treats urban trees as if they produce a series of economically valued goods, such as carbon dioxide sequestration or air pollution reduction,
2. estimates prices for these ‘goods’ (e.g. through the cost of substitutes to do the same function),
3. adds these prices together to provide the total economic ‘benefit’ provided by trees, and then subtract the costs of producing and maintaining the urban treescape.

This procedure will produce the ‘net benefit’ of urban trees to a region in financial terms. Maco and McPherson [141] followed this logic to produce a benefit-cost ratio of 3.8:1 for urban trees in the city of Davis, California, concluding that further plantings and rejuvenation of urban treescapes will produce net societal gains. Soares et al. [142] used a similar approach in Lisbon on urban street trees, arriving at a benefit-cost ratio of 4.48:1.

#### b) Qualitative approaches

Where quantitative approaches seek to gauge how the ‘magnitude’ of a specific relationship (e.g. a magnitude of preference for a particular type of tree) changes across space and across social groups, this requires that the relationship be specified by the analyst in advance. It assumes that the analyst knows which relationships are (most) important *a priori*. Qualitative approaches, in contrast, seek to understand which relationships and meanings matter to participants, be they urban residents, policymakers, scientists or activists. Such approaches seek to understand the personal and historical *meanings* of urban trees in specific urban contexts, and can include interviews, textual analysis, focus groups, participant diaries and open-ended surveys. Two examples provide an indication of the insight and utility of qualitative approaches. In the first example, Peckham et al.’s [143] semi-structured yet open ended approach to the diaries of residents in Halifax and Calgary revealed a diversity of ways in which urban trees were meaningful to participants. Some went out of their way in their commutes to walk through urban green space, and many highlighted the peacefulness of the songs of birds. In a second example, Heynen et al. [144] demonstrated the socio-economic disparity in the location and density of urban trees in Milwaukee. Owing in part to differences in capacities for tree maintenance, residents in poorer areas found urban trees to be a nuisance and a financial liability. Here, the ecosystem disservices of trees (such as infrastructure damage, fruit and leaf waste and attraction of pests, difficulties in navigation or reduced visibility, or increased economic, energy or water costs with tree management) assume more significance [144]. Planting trees in these communities would have further marginalized the views and aspirations of these communities, and certainly would not have helped lessen the environmental injustice insofar as justice relies on the disadvantaged feeling empowered and represented in urban development decisions. In both of these examples, the value of qualitative methods comes through their ability to understand the local and social-political meanings of urban trees.

While studies linking urban nature to human well-being are illuminating and valuable, care needs to be taken in making generalizations about these relationships across urban environments and across social and economic groups. Qualitative and mixed methods research in particular have demonstrated that assuming ‘positive’ relations between urban street trees and psychological well-being can be politically problematic and not just empirically unwarranted. For example, extrapolating the preferences of white middle-class urbanites to socially and economically marginal groups (as in the Milwaukee example) could be seen as ethically and politically irresponsible [144].

Clear links between the underlying processes need to be established in order to understand apparently contradictory results. For example, epidemiological cross-sectional studies, such as that of Lovasi et al. [135], found an inverse association between density of urban trees and the prevalence of childhood asthma (but not with hospitalisations due to asthma). Although the analysis controlled for some confounding factors, perhaps due to the scale of the study, clear physical, environmental or psychological mechanisms were not identified. Similarly, Donovan et al. [145] showed that a loss of trees in the neighbourhood resulted in increased mortality related to cardiovascular and lower-respiratory-tract illness, but no mechanism was suggested. Scale can also be important in interpreting apparently conflicting results in the literature. For example, regardless of the method, the evidence supporting the value of vegetation in promoting increased physical activity has produced mixed conclusions [146]. Understanding the conflict between viewing trees as a beneficial environmental feature supporting the ‘walkability’ (and hence physical activity promoting nature) of urban areas [147, 148] versus notions of reduced visibility and fear need to be understood in local neighbourhood contexts. Furthermore, the local role of environmental factors may be important as shading from tree canopies may be desirable in warmer climates but less so in cooler climates or on cold days.

#### c) Implications

What is at stake in these choices about how to model the cultural ESS produced by street trees? Clearly, the ESS literature does not provide a ‘universal list’ of cultural services, and this review suggests that practitioners should be sceptical of using one, even if one is proposed. Rather, these choices about methodological approach are about connecting ESS analysis to the political contexts and social groups who will make use of the research. The social meanings of urban trees are not pre-given or non-political; the meanings of urban trees are historical, they are symbolic, and they are differentiated across social groups. Ignoring the context of decision making can lead to outcomes that may produce net costs for many or all involved. Kirkpatrick et al. [149] highlight that planning for urban trees needs to consider the distribution and dynamics of residential ownership and regulations upon private property. Any coherent environmental justice strategy built around equitable access to urban green space needs to fully consider the dynamics driving the present and future distribution of environmental outcomes. Wolch et al. [150] further warn that strategies to increase access to urban green space for poor neighbourhoods can paradoxically result in higher property values and gentrification (displacement of poorer residents through higher rents). It is crucial then to understand the local contexts and meanings of urban street trees when conducting analyses, rather than assume that such meanings will follow the quantitative predictions derived from surveys of narrow social groups and locational contexts.

## Conclusions and recommendations

As urban greening initiatives continue to be mobilised into planning agendas and narratives of liveability, health and well-being, researchers can strengthen and shape these conversations by providing supporting inter-disciplinary analysis. Our review of ESS provided by street trees reveals that the relationships between the bio-physical properties of trees and human benefits are both complex and context-dependent. While some of the biophysical functions of trees can be summarised and described ‘in general’, the particular meanings, values and societal implications of street trees for a particular setting need to be evaluated scientifically and justified politically *in place*. Our review did not attempt to compile a master list of services and disservices for urban and street trees (for this we refer readers to Roy, et al., [2]). Rather, we have selected a number of well-known ESS for urban street trees and evaluated the extent to which these ESS relationships are in fact generalizable. Through reviewing the evidence for the ESS provided by street trees in the context of climate change, air quality and cultural ecosystem services, we conclude that the ‘benefits’ produced by street trees are shaped by various scales of biophysical context, as well as social meanings, histories and inequities that give street trees meaning to their local communities.

The challenges of translating the (physical and social) science into local policy are complex. This review demonstrates that over-emphasizing a single process in justifying urban trees (such as air pollution abatement or climate change mitigation) can have unintended consequences (such as increased pollen). The current evidence base also does not allow the impact of greening interventions to be reliably predicted from general rules or top-down frameworks. Such frameworks may support the accumulation of knowledge ‘in general’ but do not prioritise careful place-based understanding of the urban biophysical and social contexts of urban tree planting initiatives. Single-issue optimization and modelling approaches that make decisions based on the modelling of individual ‘(dis)services’ of street trees risk 1) benefiting only a small number of stakeholders, 2) reproducing relationships of power and marginality in the community, and 3) opening the potential for mal-adaptation.

Our review, in agreement with other papers in the ESS literature (e.g. Andersson et al. [151]) has also highlighted the importance of scale when determining the effect of trees on climate and health. Whilst much of the research to date has focussed on the regional and urban scale effects of vegetation on climate and health, it is much less clear what the impacts of street trees are at local scales where the result of the intervention is most clearly felt. Similarly, the net effect of individual pollutants on population health has been widely reported at regional scales, but little is known about the combined direct health effects of air pollution, pollen and temperature. This makes quantifying the resulting health impacts particularly challenging. Feedback loops also exist as a result of changes in energy consumption and carbon sequestration which can exacerbate or mitigate climate change processes.

There is a strong practitioner desire for prescriptive universal templates (which quantify the financial costs and benefits) when it comes to decision making. Institutions and governmental organisations that manage street trees often have a limited budget which requires seeking the largest possible benefit from the trees for the cost of planting, maintenance and protection of trees. Given the cost of planting initiatives and the potential lifespan of the trees, consideration also needs to be given to the expected changes in urban form and function with time and space. Clear aims are required to ensure success of a given intervention at local scale.

From our review, we argue that decision making frameworks need to be locally tailored and embedded into bottom-up decision making processes. This enables communities to articulate what matters to them about urban trees, and not just have technical scientific meanings used to justify ecological interventions (e.g. Tadaki et al. [152]). Urban greening initiatives should be pursued through a process where the multiple meanings of urban trees (cultural as well as scientific) can be articulated and deliberated together. A universal list of potential societal benefits provided by urban trees (such as those listed by Roy, et al. [2]) can provide a starting point for conversation with affected stakeholders about how urban trees might become meaningful to the future of a particular community, but scientific lists and frameworks should not be used *instead of* meaningful engagement from diverse community voices and perspectives. Frameworks such as the ‘Right Tree Right Place’ checklist for urban trees in London [153] can provide sensitizing questions that draw on accumulated scientific knowledge, while also requiring and supporting contextually specific and locally justified responses.

Where modelling is required, systems dynamics approaches could also be used to capture the complexity and dynamic interactions occurring within urban systems, and has been used previously to integrate information from different disciplines and sectors whilst maintaining a health focus. Other participatory modelling approaches which take account of different outcome goals and criteria [154–156] (within an urban area or more widely) allow the assessment of policy options and the priorities of varied stakeholders to be taken into account. Such approaches provide a practical resource which local authorities can use to guide how science can best inform policy for maximising the benefits of street trees, whilst avoiding potential maladaptation issues.

There is a clear need for in situ validation of these processes to better parameterise the underlying effects. However, attempts to seek and claim a ‘net impact’ of street trees, even for a local context, should be treated with caution. This approach implies that we know (and know how to value) all of the different effects in time and space to produce a single ‘net’ value. Finally, it is worth remembering that environmental justice concerns underlie all of these conversations about how and for whom urban greening should be done. As scientists and citizens, these opportunities to green our cities can also be seen as opportunities for creating more just social and environmental places.

This review has intended to sensitize decision makers to concerns and issues that can help develop place-specific knowledge and strategies. On the one hand, prescriptive ‘check lists’ are one useful way of accumulating and organizing knowledge about the ESS of urban trees. There remains a legitimate scientific project to compile and review accumulated knowledge about the effects of urban trees at different scales. We need to bring this knowledge together, evaluate its coherence, and assess the robustness of generalizable claims. On the other hand, simply applying generalised checklists is no substitute for meaningful policy development with diverse stakeholders about future urban environments and their meanings. We cannot assume that there are or will be robust relations across all contexts. Rather, as our review has shown, there is a need to develop reflexivity about how urban trees produce ESS for different social groups at different scales.

## Additional file

Additional file 1:
**Peer review reports.** (PDF 326 kb)
