# Supplementary material for: Health and climate related ecosystem services provided by street trees in the urban environment
Source: Environ Health. 2016 Mar 8;15(Suppl 1):36. doi: 10.1186/s12940-016-0103-6 (PMC4895605; doi:10.1186/s12940-016-0103-6)
Supplement: Additional file 1: — Peer review reports. (PDF 326 kb) [file 12940_2016_103_MOESM1_ESM.pdf]

Reviewer 1

ECOSYSTEM SERVICES PROVIDED BY STREET TREES IN THE URBAN ENVIRONMENT AND THEIR IMPLICATIONS FOR HUMAN HEALTH IN A CHANGING CLIMATE

Comments from Juliette Daniels, London Climate Change Partnership and Climate UK
29 July 2015

This paper provides a useful summary of the issues at hand regarding planting of urban street trees. The conclusion could do with tightening with regard to next steps for supporting practitioners: there is the indication that Multi Decision Criteria Analysis is the logical next step without any indication of at what spatial scale this could usefully be taken up, and without a sense of whether this is considered a job for local authorities to undertake or if further academic work could usefully support this process. I have the following specific comments to make, by line number:

45-7 – It would be useful to have a source cited here, and/or be able to know which cities this involved.

125 It would be useful to have a table with all of the benefits and disbenefits considered in the paper, as well as the ESS list of classifications.

230 - It would be helpful to the reader to express as a percentage to match the expression of green cover increase.

304-6 – Worth noting that shading will be limited in winter if the trees are deciduous. Are there any concerns regarding health impacts of leaf litter also worth mentioning here?

306-8 – Also link to light levels – health benefits of daylight in winter and Seasonal Affective Disorder.

316-19 – This is likely to be compounded by climate change for regions where more frequent and intense droughts are projected in future.

596-614 Is it possible to say something here about the tension between air quality regulation and allergen production in individual species here? Do these effects balance or cancel each other out in some cases? For example, London Plane Trees (*Platanus × acerifolia)* are cited in the iTree reports as removing the greatest load of pollutants from the air in their London survey, but they are also commonly cited as one of the main culprits for allergy-producing pollen in the city.

670-5 Can the correlation between green space and other socioeconomic factors affecting wellbeing be discounted here?

803-5 Agreed, although it would be extremely useful for practitioners if research could assist with a methodology for assessing and weighing the pros and cons of potential street tree species against one another – with so many variables (including non-health relevant such as cultural and aesthetic) to consider, city governments have their work cut out to decide which species to choose for planting programmes. Given the long term impact of such an investment decision (lifespan of trees selected, and long lead time for newly planted trees to fully provide expected services such as shading), and the universality of the challenge (city governments everywhere have to contend with this, albeit with different local variables to consider) it is an area that merits strategic support. This may only be possible at a regional level due to variability of climate, species and local needs, but could still be an efficient use of effort.

The GLA produced *A tree and woodland framework for London* (2005, Mayor of London) which suggested a 'Right Tree Right Place' checklist (p32) and a list of challenges for management of urban trees (p9) which it may be useful to refer to here. It raises positive impacts not listed in this paper, such as increased sense of privacy for city residents, as well as negative impacts, such as increased risk of subsidence, which may have an indirect impact on health and wellbeing through economic impacts to residents (as a stressor similar to flooding)
http://www.forestry.gov.uk/pdf/ltwf_full.pdf/$FILE/ltwf_full.pdf

Reviewer 2

China is now facing a fast urbanization with huge environmental changing. This has brought about many public health issues, and exacerbated with big amount of size and fast aging in population structure. As a new national urbanization strategy launched in China in 2014, it is time to review the relationship between urbanization and human health as this manuscript focused. Generally this manuscript had systemically illustrated the relationship between environmental issues and population health in context of urbanization of China and suggestions for national policies, local government regulation and individual behaviors.

There are some major issues the authors should modify before this manuscript be published.

1) In fig 1. What is the environmental health problems (or issues) refer to ? And those issues should organized with pathology of environmental risk factors exposure and give more explanation and examples for better understanding.

2) In Page 10, China has the biggest elder population in the world and recognized as a big public health issues. More, fast aging population is also a key point in China. Some other countries with aging population proportion, such as Japan. Are there any possible experience from these countries which could be referred by China? For this reason, more review on this is recommended in the text.

3) There are some blocks on fig 4. And for the corresponding text of

it, what is the correlation between the health actions at different levels? And this need more review and explanations.

More, there are some minor modification the authors should also pay attention before this manuscript be published.

1) In abstract, line 4, “a systems understanding to devise solutions…” had not be clearly stated?

2) Page 3, line 15, “Urbanization and urban expansion result in urban environmental changes, as well as residents’ lifestyle change, which can lead independently and synergistically to human health problems. In particular, uncontrolled urbanization is associated with pollution, social isolation, overcrowding…” should “independently” be “interdependently”? and “social isolation” be “social differentiation or stratification”, which bring social isolation?

3) page 4. Line 14, “and higher use of fertilizer…” should be “deeper dependence on usage of fertilizer and …”

4) page 7, Line 9, “PM2.5” the 2.5 should be subscripted, and the rest should also be changed.

5) page 7, Line 13, “Years of Life lost” should be “YLL (Years of Life Lost)”.

6) page 7, paragraph 2, line 4, “well-established” should be “well-recognized”

7) page 9, title “Climate change” should be “extreme weather” for

better expression of the context of this part?

8) page 13, line 4, “which will break the health service gap…” should be “which will make up the … gap….”?

9) page 16, there are the title for “solution at local level” and “solution at individual level” , but where is the title for the solution at national level, which is illustrated in figure 4?

In general, this manuscript had reviewed an important issue of environmental factors and public health issues under the context of fact urbanization of China. It is worth the readers referring and should be published on the special issue of “Challenges and Opportunities for Urban Environmental Health and Sustainability” in the Environmental Health journal.

Reviewer 3

URBANIZATION AND HEALTH IN CHINA, THINKING AT THE NATIONAL, LOCAL AND INDIVIDUAL LEVEL

The manuscript provides a comprehensive description of the health impact of the recent fast urbanization in China, as well as, important recommendations for public policies and urban residents, in order to, deal with those impacts. It is a well-written review paper, with sufficient number of references. However, some of them are generic. Therefore, I recommend the acceptation of the manuscript. Nevertheless, I also recommend some revisions in it before publishing. They are:

1. Revision in the abstract:
    1. The manuscript does not present the relation between the rapid urbanization in China and its impact in the international public health;
    2. The applied methodology is not clear;
    3. The results are not well linked with the introduction of the abstract.
2. Revision in the introduction:
    1. Urbanization is an important social process of human society for a long time and not only in the 21st century. This paragraph should be revised, as it is very generic. It could be eliminated and better linked with the second paragraph?;
    2. Figure 1 is not clear: e.g. “Cultivated land reduced “can impact the “food security” and induce to “Environmental health problems” or to “health problems”?.
3. Some phrases should have source:
    1. Pg.6; line 1; “China has undergone…than many other countries”;
    2. Pg.7; line 5; “Global climate change…in recent years”;
    3. Pg. 8: some other phrases are without source.
4. The source 37 is not specific for China. Nevertheless, should we consider that they are applicable to cities in China?
5. Pg. 12: Which is the difference between small cities, prefecture cities and provincial cities? In the same paragraph (Which is the “most recent statistic”?
6. Pg. 12: Last paragraph, I suggest that the authors should review the way the numbers are informed. Should they be informed without decimal number? E.g. 73% 83%.
7. Pg.15 Change the word “above” for “in this review”.
8. Figure 4: Is it applicable only for China or for cities in other countries?
9. Pg 16: How the authors consider that the “environmental and health co-benefits could be incorporated into climate policies”? Should it be considered for the mitigation and/or adaptation strategies? It is too generic.
10. Pg 17: Why “create links to urban agricultural and food policy” and not create them considering the agricultural production from rural farms?
11. If possible, I would like to have a better explanation about the Health System in China, in order to have more comprehension of the effectivity of the suggested strategies to overcome the impacts of the recent fast urbanization process in China.

Reviewer 4

Review of "Urbanization and health in China, thinking at the national, local and individual levels."

Overview: This review aims to summarize the relationships between urbanization, environmental change and health in China, and to identify actions that could be taken at national, local and individual levels on the basis of a systems understanding of the city. It succeeds in pulling together a wide range of important factors and serves as a useful reference and policy guide.

I recommend accepting this paper, but would suggest two important revisions/additions:

1) The paper would benefit from a more complete exposition of systems thinking, which, in the current text feels thrown in. A brief description of what is meant by "systems understanding" (Complex systems? Ecosystems? Governance systems? Health systems? Causal systems? All of the above?), why such an understanding is important (e.g., complexity, multidimensionality of problems, understanding co-benefits and synergies, avoidance of unintended consequences), and how this understanding is reflected in this paper would go a long way. My sense is that the authors are cognizant of these issues but haven't been explicit.
2) The set of policy options at different scales feels a bit like a laundry list, perhaps inevitable given the large number of problems that are to be addressed. My reading is that this is intended to be suggestive, a set of important examples rather than a comprehensive set of "best practices." Much of this section might therefore work better as a table of examples, with the text reserved for general principles of what types of problems should be addressed at what level(s) of governance, and how these levels interact. I would leave this decision to the authors, but the section would benefit from more structure.

A few more general points and suggested revisions:

Overall:

- The paper is well-written, but could still benefit from some English editing.

P2

- What are "information or smart cities?"
- Is the sentence on the "new features" of world urbanization needed? What does it add to the introduction?
- What is the proportion of China's urbanization from migration versus natural increase?
- What proportion of migrants are permanent residents rather than temporary/seasonal?

P3

- Can you more clearly explain what is meant by "destroy and build" in this context?
- Some of these health problems may require more explanation; e.g., social isolation and overcrowding would seem to be opposed to one another. Qualifying the statement to say "uncontrolled urbanization has been associated in some contexts with…" would help.

P4

- Figure 1 is reasonable but incomplete. Many pathways and interconnections are omitted (for example, automobiles cause road traffic injuries, noise affects mental health, public transport infrastructure affects a wide range of other factors). Nothing is said about climate change, biodiversity, urban governance, disaster risk, etc. Reverse links are not included (for example, overweight affects physical activity). The distinction between environmental health problems and human health problems isn't clear, nor are the ways in which the larger boxes are connected (e.g., lifestyle change isn't connected by a block arrow to any of the health issues). It's virtually impossible to include everything in such a figure, and I don't suggest trying to do so, but the indicative or partial nature of the diagram should be acknowledged.

P6

- It's not clear why the proportion of mortality due to complications of pregnancy, etc., is used as a contrast to major NCDs. Clearly these percentages are extremely small—wouldn't it be more relevant and interesting to show the proportion of mortality due to infectious diseases?

P7

- The title for the urban air pollution section need not include PM2.5, since other pollutants are discussed.
- For what time period and location is the WHO figure for mortality and YLL?

P9

- The climate change section should recognize the role of cities themselves in climate change. Also, development in coastal regions is a predominant feature of urbanization in China, and a major risk factor for flooding, especially during extreme events – some discussion of this is needed.

P10

- Aging is certainly a major issue in China, but it is not clear from this text how it is linked to urbanization. Please make this explicit, and also be consistent with the spelling of aging/ageing.

P12

- Please define/describe the urban categories (small cities, prefecture cities, provincial capital, municipality). How do these differ in terms of size or other characteristics?

P13

- Please round off these insurance figures (~11.9 million and ~13.7 million)
- The section on food centers around the important issue of safety, but doesn't discuss food supply, another major concern. At least a brief discussion is warranted. Also, there's not much here about the connections between urban areas, urbanization and food (e.g.,

changing diets and eating habits, increasing demand, food distribution and security, the potential role of urban agriculture, among others) .

P15

- The overview of key public health domains doesn't mention extreme events, which would seem to be important in the context of environmental change and urban risks in China.

P16

- In Figure 4 and the text, there is some conflation of the actors with the targets of action. For example, is it national or local-level governments that are responsible for providing transparent information, education, labour opportunities, etc. to individuals?
- The division of labor among levels comes across as somewhat rigid – do local authorities/municipalities have a role in setting, rather than just implementing, policy? How about individuals?
- PM25 should be PM2.5.
- Somewhere in this section on solutions, it would be good to see a brief discussion of the role of civil society.
- Some of the information in the solutions section (e.g., about polycyclic aromatic hydrocarbons or food transport) would more appropriately be in the section on the urban environment and health.
- In general, the solutions section would be easier to read if broken up into paragraphs that reflect the overarching key public health domains.

Reviewer 5

Dear Keith

Please see review below for the paper by Salmond et al. I wasn't sure if there was an online system and/or particular criteria so please let me know if so.

This paper presents a literature review of a selection ecosystem services provided by street trees. It is a very nice, well written review that provides a comprehensive summary of the evidence so far, concentrating on some of the ecosystem services (and disservices) provided by street trees with the greatest evidence base. It is a worthwhile contribution to the debate particularly as it brings the biophysical services together in one place along with the qualitative approaches. It also provides a welcome consideration of the implications of conceptualising the role of street trees in improving health and well-being through their ecosystem services and recommendations for doing this robustly. I would therefore recommend publication with the following suggestions for revision/clarification.
Line 20: 'significant economic and environmental resources' – compared with what? No vegetation at all? My understanding is that trees are considered to be relatively cost effective compared with other forms of greening. This just needs a bit of clarification.
Line 366: GUI is suddenly introduced here with no explanation/definition and then isn't mentioned again. Either remove or explain the term. Also, how is it different from UGI (Urban Green Infrastructure) which I have seen more often?
Line 379+: This section needs to make it clearer that these studies are based on modelling and using the same/similar, largely unvalidated, models so will have the same uncertainties. Again, just some clarification needed as at the moment it could be misinterpreted that these were experiments.
Line 536: 'open urban spaces' should probably be expressed as 'urban open spaces'.
Line 543: typo after 'wildlife'.
Line 557+: scientific names such as *Betula*, *Olea eropaea* etc. should be italicised and common names such as birch, olive etc. should not be capitalised unless they are proper nouns (e.g. Japanese cedar). I also feel that you should be consistent in the text between, for example, birch or *Betula* (line 591).
Line 718: This sentence should be rewritten to improve clarity.
Lines 757: I'm not familiar with this paper but I'm wondering if their findings regarding the residents in poorer areas not wanting trees was affected by the maintenance of the trees as opposed to the trees per se. Most of the negatives here (damage, fruit and leaf waste, reduced visibility etc.) are directly related to maintenance and management so if in poorer neighbourhoods the maintenance of the public realm is underfunded as is sadly often the case this may mean residents have a more negative perception of trees compared with more affluent areas where maintenance programmes might be more generous (e.g. trees are allowed to get overgrown, leaf litter is cleared away etc.). Or perhaps factors such as visibility are more of a consideration in poorer neighbourhoods where crime rates may be greater (again not related to the trees specifically as it could equally apply to street furniture or clutter obscuring sight lines). Maybe they did consider this and the poorer neighbourhoods just do not want trees but I feel this should be clarified here.

Finally, and this is a quite important point, I feel that there is something missing looking at the title '…implications for human health in a changing climate'. Much of the paper is concerned with the here and now. Yes, the role of trees in micro-climate regulation has been considered and climate change is related to the other services discussed but I don't really feel that the paper looks forward as to how these services (and disservices) will affect and be affected by changing climates. Will urban populations rely on trees more or less? How will the climate of the future impact on the ability of street trees to deliver these services (and disservices)? Is the presence of street trees going to more important for human health in the future? I think the title suggests that these questions will be discussed (perhaps not answered!) but they are not really. The paper seems to finish with a more general discussion on the decision making process regarding street trees irrespective of the changing climate (except for a mention in line 846), which is fine but should more strongly reflect the title (or change the title).

I hope this is useful feedback for yourself and the authors. Apologies for the delay and let me know if I have missed something regarding online reviewing.

Regards

Danni

Dr Danielle Sinnett
Senior Research Fellow
Centre for Sustainable Planning and Environments
University of the West of England, Bristol BS16 1QY
